# Rapid liver enlargement and hepatic failure secondary to radiographic occult tumor invasion: two case reports and review of the literature

**DOI:** 10.1186/1752-1947-6-402

**Published:** 2012-11-26

**Authors:** Christine Simone, Martina Murphy, Roger Shifrin, Tania Zuluaga Toro, David Reisman

**Affiliations:** 1 Department of Medicine, Division of Hematology/Oncology, University of Florida, 2033 Mowry Rd, Office 294, Gainesville, FL, 32611, USA; 2 Department of Radiology, College of Medicine, University of Florida, PO Box 100374, Gainesville, FL, 32610-0374, USA; 3 Department of Pathology, Immunology and Laboratory Medicine, University of Florida, Box 100275, Gainesville, FL, 32610-0275, USA

## Abstract

**Introduction:**

Unfamiliarity with certain clinical presentations, as illustrated in these cases, can lead to delayed diagnoses that in turn cause increased morbidity, prolonged hospitalization, and the need for autopsy.

**Case presentation:**

In Case 1, a 63-year-old Caucasian woman presented with hepatic enlargement and insufficiency which progressed and resulted in her death over a period of less than 2 weeks. The patient underwent a detailed workup included magnetic resonance imaging and computed tomography scan of her liver, which did not reveal the source of her liver enlargement. Due to her progressive liver enlargement and insufficiency, she developed a life-threatening esophageal variceal bleeding during her hospital stay which further delayed the attainment of her diagnosis. She finally underwent a videoscopic laparotomy and liver biopsy which revealed complete replacement and filling in of the liver sinuous with Indian filing lobular breast cancer. The patient died shortly after her diagnosis and before she could be discharged.

In Case 2, a 68-year-old Caucasian woman with non-small-cell lung cancer was admitted to our Oncology in-patient service with a presentation of rapid hepatic insufficiency and severe liver enlargement. Like the patient in Case 1, during her hospitalization, this patient underwent a thorough radiographic evaluation, including computed tomography and magnetic resonance imaging, to identify the source of her symptoms. Radiographic imaging showed only hepatomegaly and no discrete focal lesions. As the multiple imaging studies over a period of a week did not reveal a clear cause for her symptoms, she finally underwent an interventional radiology core biopsy which showed complete replacement of her liver with non-small-cell lung cancer. Her condition rapidly progressed due to continued liver enlargement and she died due to frank liver failure before her diagnosis was affirmed and she could be discharged.

**Conclusion:**

Both of these cases illustrate the potential difficulties in diagnosing liver-infiltrative malignancy and the need for a high index of clinical suspicion for occult infiltrative malignancy in the liver to determine the appropriate therapeutic intervention, including further treatment of malignancy, palliative care, or determination of candidacy for liver transplantation. Because the diagnosis for the cause of symptoms and hepatomegaly was elucidated only by liver biopsy which occurred much later in their hospital course, both patients died while in the hospital instead of at home or in a hospice. Moreover, these delays in diagnosis and development of morbidities due to the progressing liver failure further prevent any possibility of early initiation of palliative treatment. Initial recognition of this type of presentation can lead to a prompt diagnostic biopsy and diagnosis. Giving the patient a correct diagnosis is one of the fundamental goals of oncology: a goal that, as illustrated in literature review, is not always achieved. Although treatment options in such cases often may be limited, prompt discharge from the hospital and/or admission into a hospice program can potentially afford the patient the best quality of life and help protect the patient’s dignity.

## Introduction

Lung and breast cancer are common cancers that typically present with a dominant primary lesion and metastasis to one of several places, most commonly the brain, bone, liver, and the adrenal glands. Common cancers such as breast or lung present more frequently in an atypical manner than the presentation of an atypical or rare disease. As such, it sometimes takes a high index of suspicion to diagnose these cancers. When these cancers metastasize to other organs, they tend to form discrete lesions that are easily identified by standard radiographic methods, including magnetic resonance imaging (MRI) and computed tomography (CT). However, when the tumor radiographically blends with the normal organ parenchyma, the diagnosis of metastatic disease or recurrence can be difficult. Thus, extensive workup can ensue until a tissue biopsy is obtained. When the organ involved is the liver, the potential for bleeding post-procedure may make the physician disinclined to pursue the biopsy. In this report, we describe two cases involving a 63-year-old Caucasian woman and a 68-year-old Caucasian woman presenting with radiological occult metastasis to the liver to illustrate this point.

## Case presentations

### Case 1

A 68-year-old Caucasian patient with a history of breast cancer initially diagnosed 7 years prior was admitted to our Oncology service with worsening abdominal distension. She had had a mastectomy and radiation for local invasive lobular breast cancer 4 years prior, as well as elevation in liver function tests, including aspartate aminotransferase (AST) 150IU/L, alanine aminotransferase (ALT) 233IU/L, alkaline phosphatase of 734IU/L, and bilirubin of 3.4mg/dL. She first had an abdominal CT that showed a significantly enlarged liver, ascites, and no adenopathy or metastasis. An MRI of the liver also showed that the liver was essentially homogenous, with no focal lesions visible.

The patient underwent paracentesis, from which the ascites appeared to be transudative, with no cancer or infectious etiology found. In her third hospital day, she suffered an acute esophageal variceal bleed that prompted her to be admitted to the Intensive Care Unit (ICU) for urgent endoscopy and cauterization. When she returned to the floor, she then underwent a laparoscopic biopsy of the liver that showed infiltrating lobular breast cancer. Lobular carcinoma of the breast is known to grow in a so-called ‘Indian file’ pattern (single rows of cells rather than large clusters or glands). In this case, the tumor was found within vessels and liver sinusoids, giving a homogenous appearance to the patient’s prior imaging studies. The patient died shortly after diagnosis and less than 2 weeks after being admitted with liver failure.

### Case 2

The patient was a 63-year-old Caucasian woman who was initially diagnosed with Stage IIIA adenocarcinoma of the lung. Over the next 4 months, she underwent six cycles of cisplatin and etoposide. Two weeks later, her positron emission tomography (PET)-CT scan showed some mild progression of primary right upper lobe lesions as well as increased supraclavicular lymphadenopathy on the left lobe. The CT scan at that time showed a normal-sized liver, with the size measured at 13cm from dome to tip and a volume of 1194cc.

Four weeks after completing her chemotherapy, she presented to the Emergency department with a two-week history of rapidly worsening right upper quadrant pain and abdominal distension. She reported that it had hurt to cough or sneeze for that period of time. In the Emergency department, her liver enzymes were noted to be moderately elevated at aspartate aminotransferase (AST) 223IU/L, alanine aminotransferase (ALT) 156IU/L, alkaline phosphatase 883IU/L, lipase 20IU/L, total bilirubin 1.6mg/dL, and direct bilirubin 0.8mg/dL. On physical examination, the patient was extremely sensitive to palpation or percussion over the right upper quadrant and along her lower right ribs. Her liver was palpable 4 to 5cm below the costal margin in the midline. Her physical examination showed no signs of splenomegaly, caput medusae, hemorrhoid, or spider angiomas on her chest or neck. Her sclera was anicteric; her examination was otherwise unremarkable.

**Figure 1:**
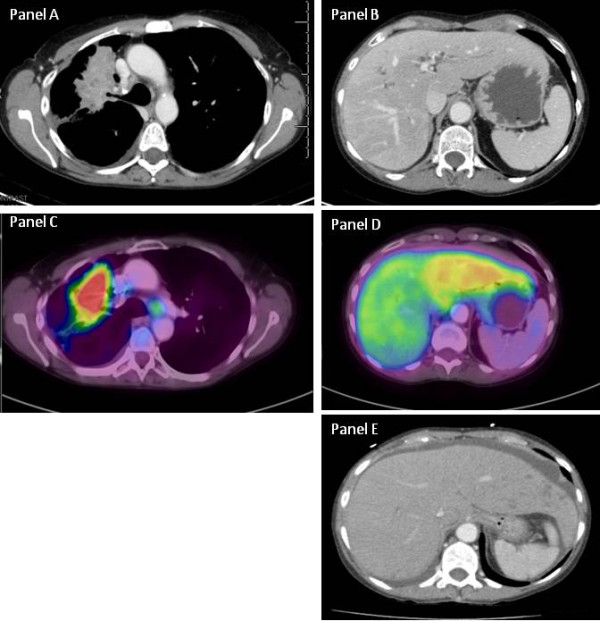
**Case 2: Computed tomography (CT) and positron emission tomography.** Panel **A**: Computed tomography of the chest shows the primary lesion in the right upper lobe caused by partial obstruction of the right upper lobe bronchus. Panel **B**: The CT scan of the liver shows essentially a normal-appearing liver without focal infiltrates present. Panel **C**: Positron emission tomography scan of the primary lesion shows intense fluorodeoxyglucose (FDG) uptake with some uptake noted in lymph nodes. Panel **D**: The right lobe of the liver looks normal, whereas the left lobe shows diffuse low FDG uptake suggestive of a pathological process. Panel **E**: A homogenous liver.

A CT scan performed in the Emergency department prior to admission, as detailed in Figure 1 (notably, less than 1 month after her prior scan) showed enlargement of both lobes of her liver; the liver was described as having a homogenous texture and no focal metastasis. Specifically, her liver on this CT study showed an impressive increase in size from 13cm to 18cm from dome to tip with a corresponding increase from 1194cc to 2655cc, which occurred over a 4-week period. Additionally, there was a rim of fluid between the capsule of the liver and parenchyma. Her gallbladder had been surgically removed. The patient was admitted to the Oncology service for further workup of the specific cause of her abdominal pain and to plan for pain control. She underwent a PET scan (panels C and D of Figure 1), which showed no areas of specific uptake indicating metastatic lesions.

**Figure 2:**
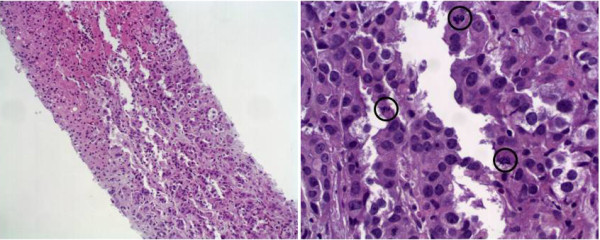
**Case 2: Core biopsy for the liver of a 63-year-old patient with a rapidly enlarging liver.** Panel **A**: Liver biopsy with metastatic adenocarcinoma. Liver tissue (top left) is diffusely infiltrated by malignant cells (bottom right) (hematoxylin-eosin stain; 10×). Panel **B**: Case 2: High-power view of tumor cells with a high nuclear-to-cytoplasmic ratio, prominent nucleoli, and frequent mitosis (black circles) (hematoxylin-eosin stain; 40×).

Given the homogeneity of the liver as evidenced by the CT scan, the patient underwent CT-guided liver biopsy, which showed diffuse infiltration by adenocarcinoma (Figure 2). Tumor cells showed strong nuclear expression of thyroid transcription factor-1, cytoplasmic expression of cytokeratin (CK)7, and were negative for CK20, indicating a primary cancer of the lung. Of interest, the biopsy only showed a small amount of uninvolved liver parenchyma, and the tumor cells demonstrated frequent mitotic figures, attesting to rapid cancer growth illustrated with the gross enlargement of the patient’s liver that had occurred over this 4-week period. With this significant finding on tissue biopsy, focus shifted to a palliative approach with control of pain in accordance with the patient’s wishes and given the futility of treatment due to the worsening hepatic failure. The patient died on the fourth day of hospital admission before her diagnosis could be affirmed because of the significant involvement of the liver parenchyma by her malignancy.

## Discussion

When a CT scan does not reveal overt signs of metastatic disease but rather a homogeneously appearing liver, as noted in both cases, it is important to have a high index of suspicion for infiltrating cancer. Delays in diagnosis can lead to additional morbidity and mortality if therapy is not initiated early enough. Because the patient in Case 2 had an active cancer, it was more obvious that her liver was impacted by the spread of her disease. However, Case 1 points to the difficulty that occurs when the history of cancer is not necessarily recent. In these cases, it is much harder to diagnose the cause of the liver enlargement, short of a biopsy. In both cases, the patients had poor performance status upon presentation due to the stretching of the liver capsule, resulting in anorexia and weight loss, and progressed rapidly to death. The condition of these patients therefore essentially prevented therapeutic interventions from being pursued. Both cases illustrate the lethality of these diagnoses and the need for prompt recognition to institute a palliative-focused approach if clinically appropriate.

Typically, metastatic disease to the liver manifests with focal lesions noted on standard imaging with CT and MRI. The differential diagnosis for an infiltrative liver process is broad and includes fatty infiltration, drug-induced hepatitis, and viral hepatitis. However, in the two cases described above, metastatic disease may present as an infiltrating malignancy without any focal lesions on radiographic imaging and must be considered in all patients presenting with occult hepatic insufficiency.

Review of the literature does describe malignancies that may present in this manner, although the cases are somewhat infrequent; Table 1 lists a summary of the cases found in the literature. Allison *et al*. described three cases in which infiltrative cancer was identified only at autopsy, which reinforces both the severity of these manifestations and the need for a high index of suspicion for this type of clinical presentation [1]. Two of these cases were infiltrating ductal carcinoma of the breast that presented with new-onset liver failure, but radiologic imaging failed to delineate any hepatic metastases. The third case had no history of carcinoma and presented with a severe thrombocytopenic purpura-like syndrome. The cause of death was not known until autopsy revealed metastatic carcinoma of the breast. In each of these cases, at autopsy, the livers on gross examination were homogeneous, firm, tan-yellow in coloration, and contained no large metastatic lesions. However, microscopically, poorly differentiated carcinoma had diffusely infiltrated hepatic sinusoids. This is a similar presentation to our first case, although our patient had lobular breast cancer, which has the propensity of being radiographically occult. Graber *et al*. reported another case of radiographic occult breast cancer involving the liver that upon biopsy was also found to be E-cadherin-negative [2]. Martelli *et al*. described the case of a 53-year-old woman who presented with rapid progressive liver failure 4 years after treatment with chemotherapy and radiation for carcinoma of the breast. Radiographic imaging showed only an enlarged liver without any metastatic lesions, but on autopsy, the liver was found to have massive neoplastic infiltration consistent with her malignancy [3]. Likewise, Goswami *et al*. reported three cases of radiographic occult breast cancer causing acute hepatic failure [4]. Nazario *et al*. have done the most extensive clinical summary of this entity involving breast cancer [5]. These authors report that in acute liver failure caused by metastatic breast cancer, the most common histologic finding is widespread intrasinusoidal infiltration of the tumor with extensive fibrous tissue, driven by a desmoplastic response in some cases. Intravascular invasion and thrombus formation impacting tissue perfusion probably facilitates hepatic necrosis commonly seen in these cases. Involvement of between 80 and 90% of hepatic parenchyma by the tumor is thought to lead to jaundice and liver failure. Similarly, two cases of radiographic occult neuroblastoma in infants have been reported, where tumor cells did not form discrete tumor masses, but on light microscopy were observed to infiltrate hepatic sinusoids [6]. Transitional cell carcinoma of the bladder has also been observed to diffusely infiltrate hepatic sinusoids as described by Lanzas Prieto [7] where ultrasound and CT imaging did not reveal nodules but rather heterogeneous areas in the liver. This patient succumbed to acute liver failure shortly after presentation; on autopsy, this patient was noted to have an enlarged liver without any nodules, and for whom microscopy revealed hepatic sinusoid infiltration by transitional cell carcinoma of the bladder. Alcalde [8] described a similar case of a 69-year-old man with a history of treated urothelial carcinoma who had presented with upper gastrointestinal (GI) bleeding. He then developed acute liver failure and died 20 days after initial presentation. In this case, imaging also failed to reveal liver lesions, but a postmortem liver biopsy again showed infiltration of hepatic sinusoids by urothelial cell carcinoma, consistent with other clinical cases discussed in this report.

**Table 1:** Summary of reported cases of radiological occult cancer infiltration of the liver

**Presenting symptoms**	**Age (years)**	**Prior history of cancer**	**Radiological test**	**Reported findings**	**Other lab test**	**Biopsy/Autopsy results**	**Reference number**
Nausea, vomiting, abdominal pain	36	Ductal carcinoma 9 months prior	CT	No abdominal lesions	Increased LFTs	Breast cancer	3
Anorexia and general fatigue	46	Mycosis fungoides	CT and US	Normal liver	20-fold increased LFTs	Mycosis fungoides	13
Mild fever with elevated LFTs	50	None	CT	Hepatomegaly	Elevated LFTs, low plts and HCT	Peripheral T-cell lymphoma	12
Abdominal pain, decreased appetite	54	None	CT and US	Hepatomegaly and ascites	Low HCT	Small-cell lung cancer	10
Worsening ascites, jaundice, and hematochezia	54	Breast cancer 9 years prior	CT US	Probable benign cysts, ascites	Elevated LFTs	Poorly differentiated adenocarcinoma	4
Altered liver test	57	None	CT and US	Heterogeneous liver parenchyma	Thrombocytopenia	Breast lobular carcinoma	5
Clinical signs of hepatocellular injury	59	Renal cell carcinoma	Liver MRI	Hepatomegaly	None reported	Renal cell carcinoma	14
Anorexia and abdominal distension	62	None	US and CT	Hepatomegaly and ascites	Elevated CA-125	Melanoma	15
Abnormal liver function	62	None	CT and MRI	Marked liver enlargement, no lesions	Increased LFT	Small-cell lung cancer	9
Acute hepatic failure	68	None	CT scan	Negative	High LFTs	Metastatic prostatic carcinoma.	16
Gastrointestinal hemorrhage	69	None	CT	No liver metastatic disease	LFT elevation	Urothelial carcinoma	8
Acute severe hepatic failure	77	T-cell lymphoma	CT and US	Homogeneous enlarged liver, no ascites	Bili 9.1, increase LFTs	Small cell carcinoma	11
Infants with hepatosplenomegaly	<1	None	CT /MRI	Liver enlargement	No biogenic amines	Neuroblastoma	6

Bili = bilirubin; CA = cancer antigen; CT = computed tomography; HCT = hematocrit; LFT = liver function test; plts = platelets; US = ultrasound; MRI = magnetic resonance imaging.

Miyaaki *et al*. describes a case of disseminated small-cell lung cancer (SCLC) that resulted in fulminant hepatic failure (FHF) [9]. Imaging by both CT and MRI of the abdomen showed marked, rapidly progressing hepatomegaly; however, no nodular lesions were visible in the liver, similar to Case 2. At autopsy, there was almost complete parenchymal replacement with metastatic tumor in the liver. Furthermore, these cases also highlight the important point that neoplastic involvement of the liver should be considered in the differential diagnosis of rapid hepatic enlargement with or without FHF of unknown etiology. Gilbert *et al*. reported an almost identical clinical scenario with another patient who presented with diffuse radiographic occult hepatic involvement that led to the rapid death of the patient without a definitive diagnosis [10]. At autopsy, the patient’s liver was grossly enlarged and weighed 5200g. It was slightly nodular and mottled in appearance but not cirrhotic. Bilirubin congestion and fatty changes were present, but the ducts were patent. Diffuse, white pinpoint plaques were noted throughout the parenchyma and by light microscopy were found to contain massive and diffuse infiltration by metastatic SCLC with concurrent hepatic necrosis.

Another case of occult SCLC involvement of the liver resulting in FHF was reported by Rajvanshi [11]. As with the previously described cases, this patient, at autopsy, was found to have on gross examination an enlarged liver weighing 3400g with a ‘salt and pepper’ distribution of metastases and no evidence of normal liver parenchyma. By light microscopy, few hepatocytes were noted and near-complete parenchyma replacement with metastatic small cell carcinoma, undifferentiated oat cell, bronchogenic type, was observed. Rajvanshi also reported a case of lymphoma involvement of the liver that was also radiographically occult and resulted in rapid patient death from hepatic failure [11]. Similarly, Kim *et al*. and Trudel *et al*. found that lymphoma can occur primarily in the liver, and in roughly 10% of these cases, it is radiographically occult and not detected until a biopsy is performed [12,13].

In Nascimento [14], a 59-year-old otherwise healthy man presented initially with expressive aphasia and visual field deficits and was subsequently diagnosed with a left bladder trigone mass. He was later determined to have renal cell carcinoma and underwent right nephrectomy. At that time, his liver appeared normal on imaging. However, he was admitted a year later for worsening renal function with elevation in hepatic enzymes. Imaging via ultrasound showed a heterogeneous, although enlarged, liver. There was increased signal of the entire liver noted on MRI. Subsequently, the patient underwent exploratory laparotomy where gross examination by the surgeon failed to reveal evidence of malignancy; however, biopsy of the liver showed metastatic renal cell carcinoma. Shan [15] describes a case of diffuse liver infiltration by melanoma of unknown primary origin in a 62-year-old woman who presented with hepatomegaly and ascites; her CT and MRI scans demonstrated hepatomegaly without any focal lesions. She had a comprehensive workup for malignancy. Finally, subsequent liver biopsy, as her prior extensive workup was unrevealing, was found to contain intrasinusoidal infiltration by malignant melanoma cells with an unknown primary.

When hepatic involvement occurs with tumors, the degree of hepatic enzyme elevation varies upon presentation, but is often found to correlate with the degree of intrasinusoidal hepatic involvement. In severe cases with rapidly growing tumors, patients can present with FHF and subsequent multisystem organ failure as a result of homogenous infiltration of the liver by metastatic disease. Shakir [16] presented a case of metastatic prostate adenocarcinoma in which a 68-year-old man presented with FHF. An abdominal CT scan revealed hepatomegaly without focal lesions, and Doppler ultrasound showed hepatomegaly and hepatic steatosis. The patient’s condition rapidly declined, with multiorgan system failure necessitating emergent ICU interventions. Autopsy revealed an enlarged liver weighing 3478g, with the biopsy showing intravascular and intraparenchymal widespread metastatic prostatic adenocarcinoma.

Common to all of these cases is that both CT and MRI imaging failed to diagnose metastatic disease, as no discrete masses or focal lesions were readily identifiable. In all instances, a biopsy was necessary to confirm the diagnosis of infiltrative metastatic disease. This is important because FHF is frequently caused by etiologies other than cancer: typically, and in the majority of patients, by viral hepatitis or drug toxicities. In 20% to 40% patients with FHF, no cause is determined, despite extensive exhaustive workup. As liver transplantation can provide long-term benefit for patients, it is important to reach a definitive diagnosis and identify the cause, since liver transplantation is contraindicated in malignant infiltration of the liver because these patients will develop further metastatic disease after transplantation [17]. Rojter *et al*. provided a testament to this situation in their review, where three patients were referred for liver transplantation after negative radiographic workup via CT and ultrasound for liver failure. In each case, before or after liver transplantation, biopsy revealed that each case had vascular tumors present in the liver, either angiosarcoma or hemangioendothelioma [18]. In these cases, where vascular tumors were involved, MRI can help with the diagnosis, as two of the three cases were suspected to have tumor burden in the liver after MRI imaging. Radiographically undetected involvement of abdominal organs, including the liver, is not uncommon with many different tumor types, as summarized in Table 1. Winkelmann [19] conducted a correlation study of abdomen CT imaging with autopsy findings in patients with malignant tumors, finding that CT scans missed between 5 and 10% of metastatic lesions. This is confirmed in this report, as breast cancer, lung cancer, and sarcomas can massively involve the liver but remain radiographically occult. Unfortunately for many of these patients, these critical biopsies and subsequent diagnosis had to await postmortem examination, given the rapid progression of malignancy resulting in death. This may be inconsequential, as this overwhelming tumor involvement of the liver is inevitably fatal.

## Conclusion

This case series highlights the not-infrequent occurrence of radiographic occult cancers in the liver that can present to both the family medicine practitioner and the seasoned oncologist.

Experience and familiarity with this type of presentation can foster the necessary high index of clinical suspicion that can prevent misdiagnosis in patients who present with hepatomegaly, occult hepatic insufficiency, or rapidly progressing pain without an obvious explanation. If this type of presentation is initially recognized, a diagnostic biopsy can quickly occur and help overcome the clinical hesitation to biopsy a swollen and enlarged liver. It also helps avoid the need for an autopsy, which unfortunately is all too common, as demonstrated by our literature review, where many diagnoses were only made postmortem. Moreover, not deferring or delaying a diagnostic biopsy can prevent undue clinical deterioration due either to the rapid progression of the untreated cancer or the cancer’s associated morbidity, such as GI bleeding, leading to prolonged hospital stays and admission to the ICU. Since aggressive treatment-based options are often poorly tolerated and ineffective in such cases, transition to an outpatient palliative approach and comfort care is often warranted, as evidenced in Case 2, if pain control can be achieved. In summary, then, quick recognition of this diagnosis and confirmatory biopsy will usually lead to the best available outcome for the patient.

## Consent

Written informed consent was obtained as proscribed by the University of Florida Institutional Review Board (IRB) from either the patient’s or the patients’ next of kin for publication of this manuscript and accompanying images. A copy of the written consents are available for review by the Editor-in-Chief of this journal. This case report follows the guidelines and policies of the University of Florida IRB.

## Competing interests

The authors declare that they have no conflict of interests.

## Authors’ contributions

CS and MM wrote, drafted, and edited the manuscript. TTZ analyzed, reviewed, and provided the pathology section and photographs. RS analyzed, reviewed and provided the radiologic information and pictures. DR initially drafted, revised, and oversaw the development of this manuscript. All authors read and approved the final manuscript.
